# Fluorescein Derivatives as Antibacterial Agents Acting via Membrane Depolarization

**DOI:** 10.3390/biom10020309

**Published:** 2020-02-15

**Authors:** Pavel A. Nazarov, Roman S. Kirsanov, Stepan S. Denisov, Ljudmila S. Khailova, Marina V. Karakozova, Konstantin G. Lyamzaev, Galina A. Korshunova, Konstantin A. Lukyanov, Elena A. Kotova, Yuri N. Antonenko

**Affiliations:** 1 Belozersky Institute of Physico-Chemical Biology, Lomonosov Moscow State University, 119991 Moscow, Russia; kirsanov_roman@mail.ru (R.S.K.); khailova@genebee.msu.ru (L.S.K.); lyamzaev@gmail.com (K.G.L.); korsh@genebee.msu.ru (G.A.K.); antonen@genebee.msu.ru (Y.N.A.); 2 Department of Biochemistry, University of Maastricht, Cardiovascular Research Institute Maastricht, Universiteitssingel 50, 6229 ER Maastricht, The Netherlands; s.denisov@maastrichtuniversity.nl; 3 Center of Life Sciences, Skolkovo Institute of Science and Technology, 121205 Moscow, Russia; mvk752002@gmail.com (M.V.K.); kluk@ibch.ru (K.A.L.)

**Keywords:** fluorescein derivative, antibacterial agent, bacterial membrane depolarization, mitochondria, membrane potential, fluorescent uncoupler

## Abstract

Appending a lipophylic alkyl chain by ester bond to fluorescein has been previously shown to convert this popular dye into an effective protonophoric uncoupler of oxidative phosphorylation in mitochondria, exhibiting neuro- and nephroprotective effects in murine models. In line with this finding, we here report data on the pronounced depolarizing effect of a series of fluorescein decyl esters on bacterial cells. The binding of the fluorescein derivatives to *Bacillus subtilis* cells was monitored by fluorescence microscopy and fluorescence correlation spectroscopy (FCS). FCS revealed the energy-dependent accumulation of the fluorescein esters with decyl(triphenyl)- and decyl(tri-p-tolyl)phosphonium cations in the bacterial cells. The latter compound proved to be the most potent in suppressing *B. subtilis* growth.

## 1. Introduction

**Figure 1:**
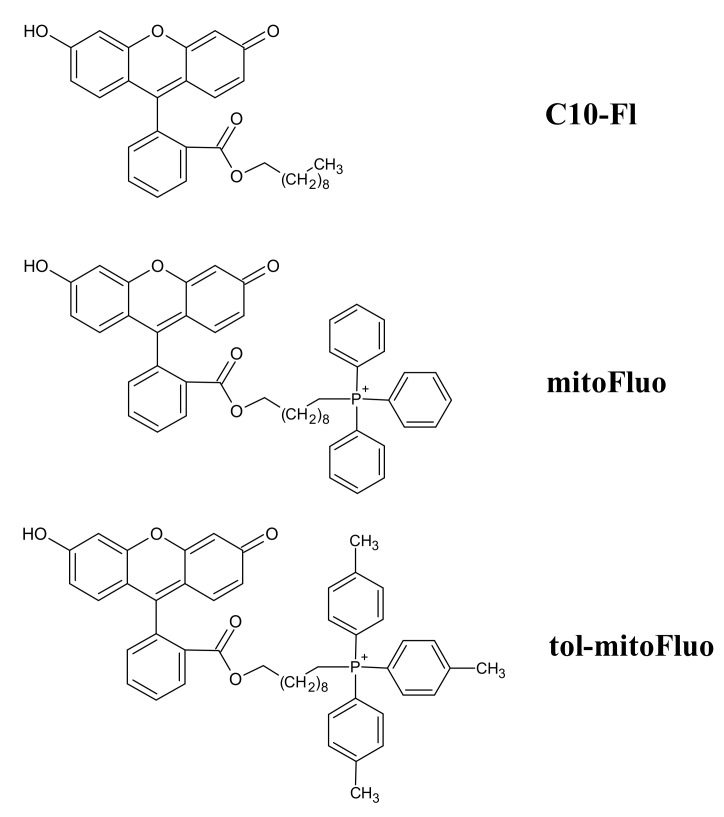
The chemical structures of tol-mitoFluo (fluorescein decyl(tri-p-tolyl)phosphonium ester), mitoFluo (fluorescein decyl(triphenyl)phosphonium ester), and C10-FL (fluorescein decyl ester).

In the 60s–70s of the last century, small molecules that uncouple respiratory and photosynthetic electron transfer from ATP synthesis, briefly called mitochondrial uncouplers, served as crucial tools in deciphering the mechanistic basics of cell bioenergetics, namely, the role of the proton motive force as the major intermediate in energy storage. The main arguments in favor of Mitchell’s chemiosmotic theory were based on the ability of uncouplers to cause the collapse of the membrane potential by carrying protons across membranes. At present, there is a revival of interest in uncouplers because of the growing evidence of their broad-spectrum therapeutic efficacy [1,2,3,4,5,6,7,8,9], including antibacterial potency [10,11,12,13,14,15,16,17,18,19], which was actually found very early [20]. To this end, a series of esters of fluorescein were obtained and studied at our laboratory, manifesting themselves as rather effective mitochondrial uncouplers [21,22,23]. Amongst them, the most intriguing compound was fluorescein decyl(triphenyl)phosphonium ester (mitoFluo, Figure 1), which was able to accumulate in energized mitochondria due to a cationic triphenylphosphonium (TPP) moiety, which was readily monitored via its bright fluorescence [22], and which exhibited neuro- and nephroprotective properties [23]. Our data revealed the necessity of a long alkyl linker between the cationic moiety and the proton-dissociating (fluorescein) fragment to render mitoFluo a mitochondria-targeted uncoupler [22,23]. To further improve the membrane permeability of mitoFluo, thereby enhancing its membrane potential-driven accumulation in mitochondria, in this study we synthesized fluorescein decyl(tri-p-tolyl)phosphonium ester (tol-mitoFluo, Figure 1). Earlier, the introduction of methyl substituents into phenyl rings of TPP was shown to augment the membrane permeability [24], accumulation in mitochondria [25], and antibacterial potency of TPP derivatives [26].

The present study is focused on the antibacterial activity of fluorescein-derived protonophoric uncouplers. In particular, tol-mitoFluo, mitoFluo, and fluorescein decyl ester (C10-FL) appeared to be potent antibacterial agents depolarizing the bacterial membrane, with tol-mitoFluo being the most effective of them in inhibiting bacterial growth.

## 2. Materials and Methods

### 2.1. Materials

Components of the bacterial Luria-Bertani (LB) media were purchased from Helicon Company (Moscow, Russia). Mueller-Hinton (MH) medium was purchased from HiMedia Laboratories (Mumbai, India). Other reagents were from Sigma-Aldrich (St. Louis, MO, USA).

### 2.2. Synthesis of mitoFluo, tol-mitoFluo and C10-FL

The synthesis of mitoFluo (Figure 1), 10-[2-(3-hydroxy-6-oxo-xanthen-9-yl)benzoyl]oxydecyl(triphenyl)phosphonium bromide, was performed according to the previously described procedure [22], via the quaternization of triphenylphosphine and then the synthesis of fluorescein ester by reaction with the resulting 10-bromodecyl(triphenyl)phosphonium bromide. Similarly, the synthesis of tol-mitoFluo (Figure 1), 10-[2-(3-hydroxy-6-oxo-xanthen-9-yl)benzoyl]oxydecyl(tri-p-tolyl)phosphonium bromide, involved the quaternization of tri(p-tolyl)phosphine and then the synthesis of fluorescein ester by reaction with the resulting 10-bromodecyl(tri-p-tolyl)phosphonium bromide (Scheme S1, SI). To synthesize fluorescein decyl ester (C10-FL), we used the esterification of the carboxyl group with a C10 alkyl substituent (Scheme S2, SI). The synthesis was performed via the reaction between 1-bromodecane and the previously obtained Na salt of fluorescein, i.e., by using an approach developed earlier for the synthesis of alkylrhodamine [27] and alkylfluorescein [21] derivatives. 

#### 2.2.1. Synthesis of 10-Bromodecyl(tri-p-tolyl)phosphonium Bromide

A solution of 1,10-dibromodecane (0.67 g, 2.2 mmol) and tri(p-tolyl)phosphine (0.25 g, 0.82 mmol) in benzene (0.5 mL) was heated at 80 °C during 4 d in a tightly closed flask. After completing the reaction, the reaction mass was cooled at 20 °C and evaporated to dryness. The residue obtained was dissolved in a minimal volume of dichloromethane, after which an excess of hexane was added and the suspension formed was kept at 4 °C until the solution became clear. Then, the liquid phase was decanted, the residue was dissolved again in dichloromethane, and was treated with hexane to complete precipitation. This procedure was repeated three times. Finally, the residue was dissolved in a minimal volume of the solvent system methanol-chloroform (1:6) and applied to a chromatographic silica gel column (MN Kieselgel 60, 70–230 mesh) in the same solvent system as an eluent. Detection was carried out with the help of TLC by UV-absorbance and Dragendorf reaction. Fractions with the same chromatographic mobilities were combined and evaporated in vacuo to yield 200 mg (40%) of the target compound as slightly yellow oil.

LC-MS: *m/z*: found 525.21, required 525.21.

#### 2.2.2. Synthesis of 10-[2-(3-Hydroxy-6-oxo-xanthen-9-yl)benzoyl]oxydecyl(tri-p-tolyl)phosphonium Bromide (tol-mitoFluo)

A triethylamine (60 mg, 0.6 mmol) was added to the suspension of fluorescein (117 mg, 0.35 mmol) in methanol. The mixture was stirred for 10 min and evaporated to dryness in vacuo. The residue was dissolved in DMF (3.5 mL), and the solution of 10-bromodecyl(tri-p-tolyl)phosphonium bromide (200 mg, 0.33 mmol) was added in a minimal volume of DMF. The reaction was carried out for 10 h under stirring at 70–80 °C. Then, the reaction mixture was cooled and diluted with 10 mL of dichloromethane. The organic solution was washed three times with water and was dried over sodium sulfate. The organic phase was filtered and evaporated in vacuo. The residue was dissolved in a minimal volume of the solvent mixture methanol-chloroform (1:4) and applied to a silica gel column (MN Kieselgel 60, 70–230 mesh) in the same solvent system used as an eluent for chromatographic purification. Fractions were detected by the direct visualization of colored zones and were additionally analyzed with the help of thin layer chromatography, with those having equal chromatographic mobilities being combined. The solvents were removed, and the residues were dried in vacuo. The target compound was further purified in two steps on the silica gel column (MN Kieselgel 60, 70–230 mesh) using dichloromethane-methanol-acetic acid (100:10:1) as an eluent at the first step and isopropanol:25% aqueous ammonia:water (14:1:5) as an eluent at the second step. For both steps, fractions were detected in the same manner as above. The target compound was eluted as an orange band, and 8 mg (2.8%) of orange powder was obtained after the evaporation of the solvents.

MALDI: *m*/*z*: found 775.5, required 775.35.

### 2.3. Isolation of Rat Liver Mitochondria

Mitochondria were isolated from rat liver by using differential centrifugation [28], according to a slightly modified procedure previously described [29]. The animals were handled and the experiments were performed in accordance with the international guidelines for animal care and use and the Institutional Ethics Committee of A.N. Belozersky Institute of Physico-Chemical Biology at the Lomonosov Moscow State University approved them (protocol #3 on February 12, 2018).

### 2.4. Measurement of Mitochondrial Membrane Potential and Respiration

The respiration of isolated rat liver mitochondria (RLM) was measured at the mitochondrial protein concentration of 0.8 mg/mL by using a Clark-type oxygen electrode (Strathkelvin Instruments, Motherwell, UK), as described previously [29]. 

The mitochondrial membrane potential ΔΨ was evaluated from the difference in the absorbance at 555 and 523 nm (ΔA) of the safranine O dye [30], measured with an Aminco DW-2000 spectrophotometer, as described previously [29]. Mitochondria were incubated in medium containing 250 mM sucrose, 5 mM MOPS, 0.5 mM KH_2_PO_4_, 1 mM EGTA, 2 μM rotenone, 5 mM succinate (pH 7.4), 1 μg/mL oligomycin, and 15 μM safranine O at the mitochondrial protein content of 0.6–0.9 mg protein/mL.

### 2.5. Bacterial Strains

The standard laboratory strains *Bacillus subtilis* subs. *subtilis* Cohn 1872, stain BR151 *(trpC2 lys-3 metB10),* and *Escherichia coli* Castellani and Chalmers 1919, strain W3110 *(F- lambda-IN(rrnD-rrE)1 rph-1)* were used. Staphylococcus aureus was received from the microorganisms collection of Lomonosov Moscow State University (No. 144). *Bacillus subtilis* PY79 was provided by E.A. Kubareva (Belozersky Institute of Physico-Chemical Biology, Moscow State University). *Bacillus pumilus* NCTC 8241 was provided by O.V. Efremenkova (FSBI Gause Institute of New Antibiotics). The bacterial cells were grown at 30 °C or 37 °C in LB or MH medium at the 140 rpm shaking frequency.

### 2.6. Growth Suppression Assay and MIC Determination

The growth suppression assay was performed by inoculating 200 µL of bacterial cultures (~5 × 10^5^ cells/mL) into 96-well plates (Eppendorf AG, Hamburg, Germany). The compounds were diluted in a 96-well microtiter plate to final concentrations ranging from 0.01 to 1000 µM tol-mitoFluo, mitoFluo, C10-FL, FCCP, CCCP, and DNP in a 250-mL aliquot of the total volume. The bacteria were allowed to grow for 18 or 21 h at 30 °C or 37 °C. 

MICs, the lowest concentrations that completely inhibited the bacterial growth, were determined by Mueller-Hinton broth microdilution, as recommended by CLSI, using in-house-prepared panels.

Bacterial growth was observed visually alongside with CFU and OD measurements [31]. Optical densities at 620 nm were obtained by using a Thermo Scientific Multiskan FC plate reader with an incubator (Thermo Fisher Scientific, USA). Experiments were carried out in triplicate.

### 2.7. Bacterial Depolarization

The membrane potential in *B. subtilis* was estimated by measuring the fluorescence of the potential-dependent probe DiS-C3-(5) [31,32,33,34]. *B. subtilis* from the overnight culture were seeded into fresh LB medium, followed by growth for 24 h until reaching the optical density 0.8 at 600 nm. Then, the bacteria were diluted 20-fold in a buffer containing 100 mM KCl, 10 mM Tris, pH 7.4. The fluorescence was measured at 690 nm (excitation at 622 nm) by using a Fluorat-02-Panorama fluorimeter.

### 2.8. Bacterial Permeabilization

The violation of the bacterial membrane integrity was evaluated by using the cell-impermeant nucleic acid stain TO-PRO-3 iodide [35].

### 2.9. Fluorescence Microscopy

#### 2.9.1. Agarose Pads Preparation

2% low-melting agarose in medium was boiled and then cooled to 42 °C. An agarose pad was cast by depositing 1 mL of the agarose on a 22 × 22 mm cover glass and floating another 22 × 22 mm cover glass on top. The pad was allowed to solidify for 45 min, and then one cover glass was removed. We cut agarose pads into smaller individual pads with a scalpel, creating two pads for each strain [36].

#### 2.9.2. Sample Pads Preparation

Overnight bacterial cells cultures were diluted 1:10 by fresh LB media. Bacteria cell suspensions were incubated with 2 μM mitoFluo, tol-mitoFluo, and C10-FL for 5 min and 5–7 μL loaded on top of the pads. After drying (approximately 15 min), the pads were flipped and transferred to an imaging 35 mm confocal glass bottom dish SPL 100350 (SPL Life Sciences Co, Pocheon-si, Korea).

#### 2.9.3. Equipment Setup

To study the distribution of the fluorescein derivatives in *B. subtilis* cells, we used a setup based on the inverted motorized BZ-9000 BioRevo fluorescence microscope Keyence (Itasca, IL, USA.) equipped with an HC PL Apo 100x1.40 oil lens (Nikon, Tokyo, Japan), GFP filter cube (excitation filter 470/40, emission filter 525/50), and temperature control chamber. The images were processed using FIJI ImageJ distribution [37,38].

### 2.10. Fluorescence Correlation Spectroscopy

The fluorescence correlation spectroscopy (FCS) measurements were carried out with a homemade FCS setup [29,39] including an Olympus IMT-2 inverted microscope with a 40x, NA 1.2 water immersion objective (Carl Zeiss, Jena, Germany). A Nd:YAG solid state laser was used for excitation. The fluorescence that passed through an appropriate dichroic beam splitter and a long-pass filter was imaged onto a 50-μm core fiber coupled to an avalanche photodiode (PerkinElmer Optoelectronics, Fremont, CA). The signal from an output was correlated by a correlator card (Correlator.com, Bridgewater, NJ). The data acquisition time was 30 s. The experimental data were obtained under stirring conditions which increased the number of events by about three orders of magnitude, thus substantially enhancing the resolution of the method. The peak intensities of the fluorescence traces with a sampling time of 25 μs were analyzed using WinEDR Strathclyde Electrophysiology Software designed by J. Dempster (University of Strathclyde, UK). The software, originally designed for the single-channel analysis of electrophysiological data, enables one to count the number of peaks [n(F>F_0_)] of the FCS signal having amplitudes higher than the defined value F_0_. A program of our own design with a similar algorithm (coined Saligat; provided on request) was also used.

### 2.11. Experiments with Human Cell Line Rko

Human colon carcinoma cell line RKO (ATCC CRL-2577) was cultured in Dulbecco’s modified Eagle’s medium (DMEM) supplemented with 10% fetal calf serum, streptomycin (100 U/mL), and penicillin (100 U/mL). The viability of the cells was assayed using CellTiter-Blue Reagent (Promega). The cells were seeded in 96-well plates at 100 µL/well and cultured for 24 h at 37 °C. The cells were treated with mitoFluo for 17 h, then Cell Titer-Blue (20 µL/ well) was added, and the cells were incubated for 1 h before recording the fluorescence (λ_ex_ = 560 nm; λ_em_ = 590 nm) with a Fluoroskan Ascent Microplate Fluorometer (Thermo).

## 3. Results and Discussion

**Figure 2:**
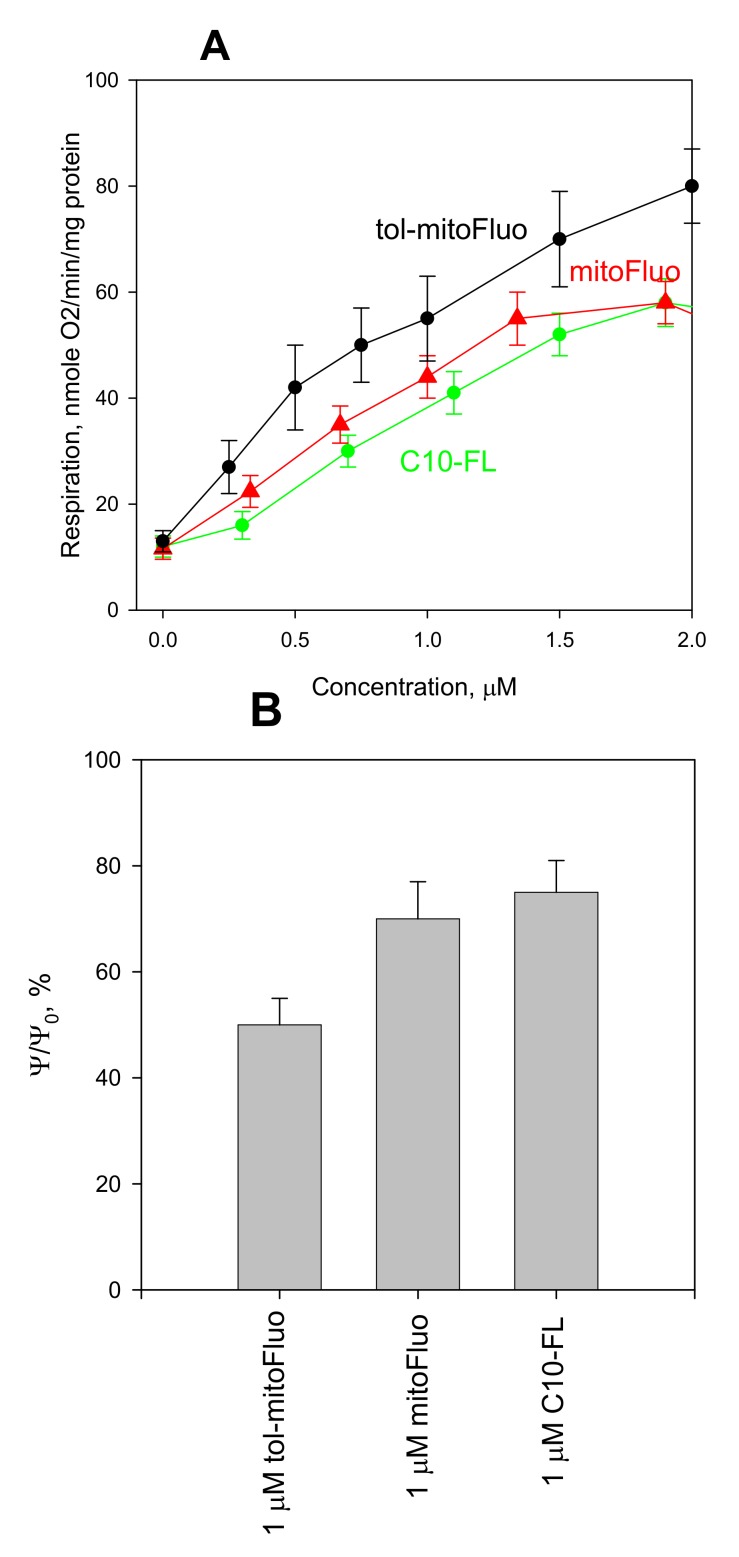
(**A**) The dose dependence of the stimulation of rat liver mitochondria respiration by tol-mitoFluo (black curve), mitoFluo (red curve), and C10-FL (green curve) with succinate as a substrate. (**B**) The effect of tol-mitoFluo, mitoFluo, and C10-FL (concentration, 1 µM) on the membrane potential of RLM estimated by the absorbance changes of the potential-sensitive dye safranine O (15 µM). Measurements of the membrane potential and respiration were performed in the incubation medium (see **Experimental Section**) with 5 mM succinate and 2 µM rotenone. The data are the mean±SD of at least three measurements.

To assess the potency of new fluorescein derivatives in dissipating the membrane potential, we probed them in a standard bioenergetics model, namely, in isolated rat liver mitochondria (RLM). As seen in Figure 2, tol-mitoFluo stimulated mitochondrial respiration (Figure 2A) and also caused the dissipation of the mitochondrial membrane potential, as measured by the absorbance changes of the potential-sensitive dye safranine O (Figure 2B), at submicromolar concentrations, being more potent than mitoFluo and C10-FL. Thus, tol-mitoFluo appeared to be an efficient mitochondrial uncoupler.

**Figure 3:**
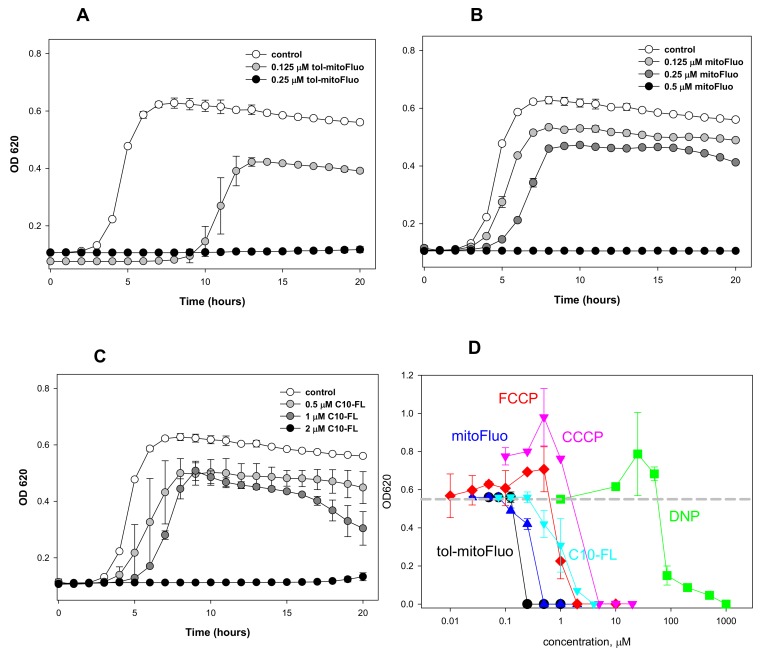
(**A**–**C**) The dose-dependent effect of (**A**) tol-mitoFluo, (**B**) mitoFluo, or (**C**) C10-FL on the growth of *Bacillus subtilis* cells. The *B. subtilis* growth was evaluated by the absorbance at 620 nm at 37 °C with a 96-well Multiskan FC Microplate Reader. (**D**) OD620 measurements of *B. subtilis* cells 20 h after the treatment with different concentrations of tol-mitoFluo, mitoFluo, C10-FL, FCCP, CCCP, and DNP. The gray dashed line shows the level of cell growth in control. The data points represent the mean±SD of at least three experiments.

In experiments with *Bacillus subtilis*, tol-mitoFluo demonstrated a high antibacterial efficacy, completely suppressing bacterial growth at a concentration of 250 nM (Figure 3A), whereas the same result was obtained with mitoFluo at a concentration of 500 nM (Figure 3B) and with C10-FL at a concentration of 2 μM (Figure 3C). The same relative efficacy of the fluorescein derivatives is revealed via a comparison of the MIC values, as shown in Table 1. Figure 3D presents the concentration dependences of the inhibiting effect of the fluorescein derivatives on the bacterial growth, as compared to those for the conventional uncouplers carbonyl cyanide p-(trifluoromethoxy)phenyl hydrazone (FCCP), carbonyl cyanide m-chlorophenyl hydrazone (CCCP), and 2,4-dinitrophenol (DNP). One can see that both tol-mitoFluo and mitoFluo appeared to be more potent antibacterial agents than all the classical uncouplers studied here. 

As seen in Table 1, the antibacterial efficacy of the fluorescein derivatives proved to be similar for several gram-positive bacteria, such as *B. subtilis* strains PY79 and Br151, *B. pumilus,* and *S. aureus*, whereas the Gram-negative bacterium *E. coli* was rather resistant to these compounds. Only high mitoFluo and t-mitoFluo concentrations (more than 15 μM) exerted a significant effect on *E. coli* growth, but we were unable to determine the MIC precisely because of the high absorbance of these dyes. We believe that similar to the previously reported data on SkQ1 (a decyltriphenylphosphonium cation conjugated to a plastoquinone moiety), the resistance of *E. coli* cells to mitoFluo could be due to its efflux performed by multiple MDR pumps, including the major *E. coli* MDR pump AcrAB-TolC [29,40].

**Figure 4:**
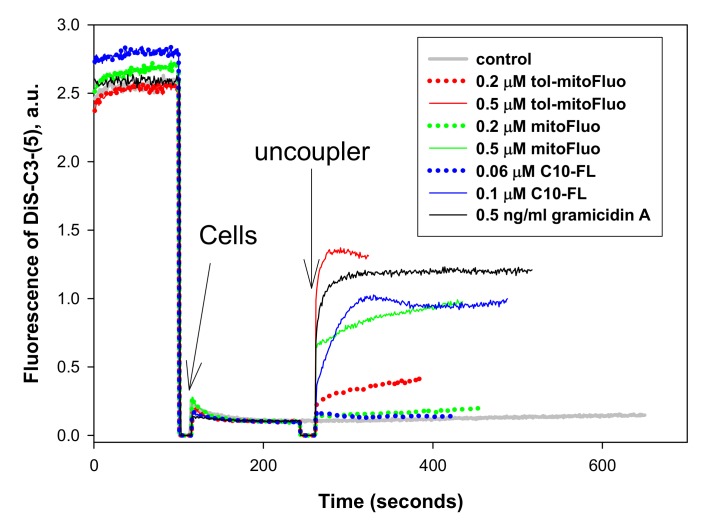
The effect of tol-mitoFluo, mitoFluo, and C10-FL on the membrane potential in *B. subtilis*. Changes in the membrane potential were monitored by measuring the fluorescence of DiS-C3-(5) (10 µM) in PBS buffer. Gramicidin A concentration, 0.5 ng/mL.

Bearing in mind the uncoupling properties of the lipophilic fluorescein esters (Figure 2), we examined their effect on the bacterial membrane potential as measured by the fluorescence changes of the potential-sensitive dye Dis-C3(5). As seen in Figure 4, all of the three fluorescein derivatives caused bacterial membrane depolarization, with C10-FL being somewhat more effective than mitoFluo and tol-mitoFluo. 

**Figure 5:**
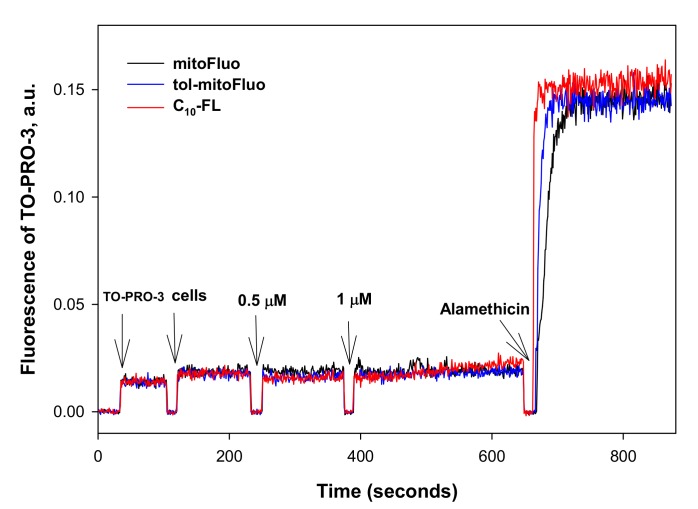
The effect of tol-mitoFluo, mitoFluo, and C10-FL (0.5 and 1.5 µM each) on the membrane permeabilization in *B. subtilis*. The changes in the membrane permeabilization were monitored by measuring the fluorescence of TO-PRO-3 (1 µM) in PBS buffer. The channel former alamethicin was added at the end of each recording as a positive control (concentration, 10 µg/mL).

To exclude the bacterial membrane permeabilization as a possible alternative reason for the depolarization, we measured the fluorescence response of the cell-impermeant nucleic acid stain TO-PRO-3 iodide [35]. As seen in Figure 5, all of the three fluorescein derivatives produced no detectable response of TO-PRO-3 iodide, whereas alamethicin, used as a positive control, exhibited a pronounced increase in the fluorescence. Based on these results, it could be concluded that tol-mitoFluo, mitoFluo, and C10-FL dissipated the bacterial membrane potential via transmembrane proton shuttling.

**Figure 6:**
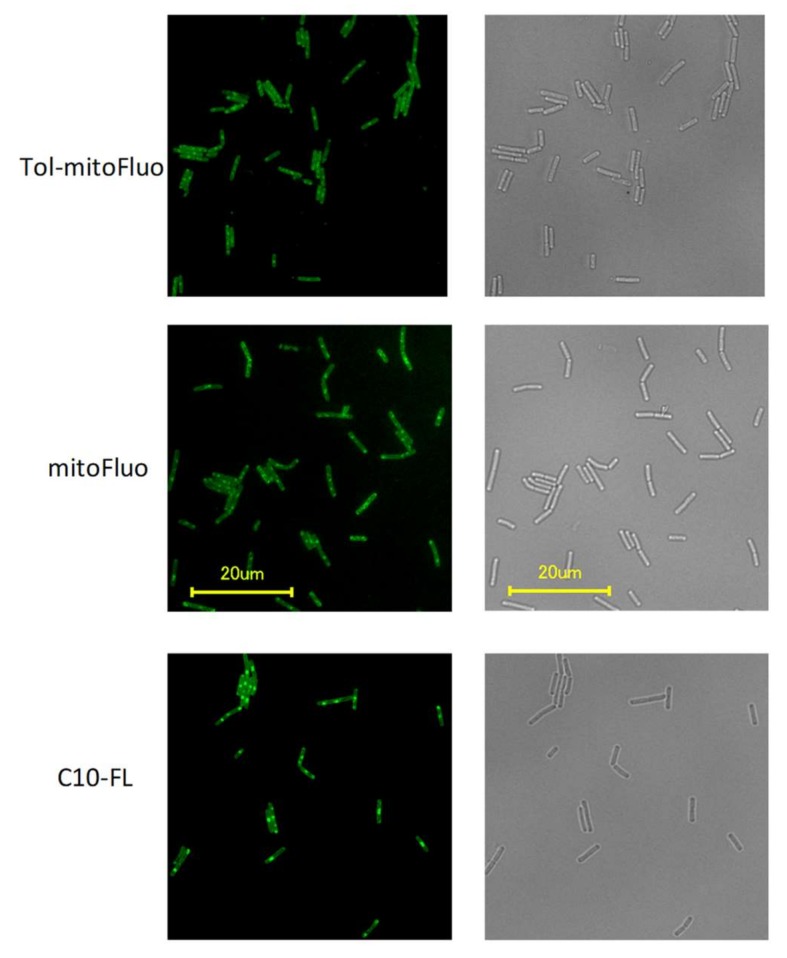
Fluorescence from *B. subtilis* cells stained with tol-mitoFluo (top row), mitoFluo (middle row), and C10-FL (bottom row), and the corresponding transmission light images. The cells were incubated with 5 μM of the dyes for 5 min, washed with LB and imaged.

To visualize the interaction of the fluorescein derivatives with bacterial cells, we employed fluorescence microscopy. The bright green fluorescence of *B. subtilis* cells incubated with C10-FL, mitoFluo, or tol-mitoFluo (5 µM of each compound, Figure 6) showed the effective accumulation of these dyes in the bacterial cells.

**Figure 7:**
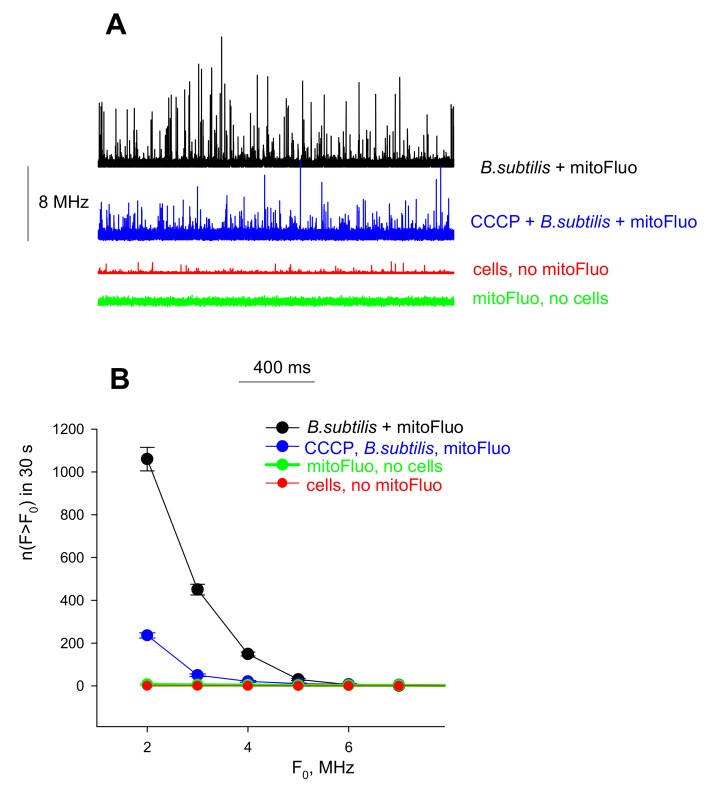
The effect of CCCP (10 µM) on the accumulation of mitoFluo by *B. subtilis* cells, monitored by FCS. (**A**) The fluorescence intensity traces of mitoFluo (100 nM) recorded with the FCS set-up in the presence or absence of bacterial cells (10^6^ per ml of PBS). (**B**) The corresponding dependences of the number of peaks with the fluorescence intensity F exceeding the threshold F_0_, *n*(F > F_0_), on the value of F_0_.

The binding of mitoFluo to *B. subtilis* cells was also measured with fluorescence correlation spectroscopy (FCS) at low concentrations of C10-FL, mitoFluo, or tol-mitoFluo (100 nM), providing a vigorous statistical analysis of the binding. The FCS setup recorded fluctuations in the emission signal of a small number of fluorescent particles diffusing into and out of the focus volume of the excitation laser (about 10^−15^ L). The measurements were conducted under the conditions of stirring the solution in order to improve the statistics of the peak measurements. The mitoFluo solution without bacterial cells gave a fluorescence signal with fluctuations of very low amplitudes (Figure 7A, green curve), because there was a large number of free mitoFluo molecules in the confocal volume. If *B. subtilis* cells were incubated in the presence of mitoFluo, the fluorescence recording contained peaks of high amplitude (Figure 7A, black curve), which corresponded to the appearance of cells bearing a large number of mitoFluo molecules. Upon the addition of CCCP, i.e., under the conditions of cell membrane depolarization, the high-amplitude peaks became less frequent (Figure 7A, blue curve), thus confirming a decrease in the number of stained cells in the presence of CCCP. Figure 7B presents the plots of the number of peaks detected during 30 s versus the amplitude. Thus, the FCS method showed the mitoFluo accumulation in *B. subtilis* cells upon energization of the bacterial membrane. Similar results were obtained with tol-mitoFluo (data not shown).

**Figure 8:**
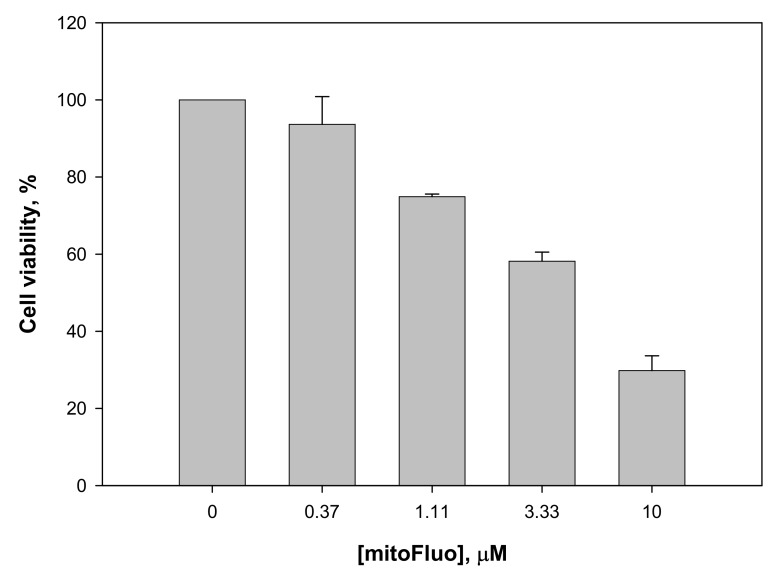
The viability of Rko cells in the presence of mitoFluo. Rko cells were incubated with mitoFluo for 17 h. The cell viability was determined with the Cell Titer-Blue reagent (Promega). The data are the mean±SD of at least three measurements.

In view of the antibacterial activity of the fluorescein derivatives observed in the present study, it was important to estimate their cytotoxicity for mammalian cells. For this purpose, we used the human colon carcinoma Rko cell line. In the MIC concentration range, no significant cytotoxic effect of mitoFluo on the Rko cells was detected (Figure 8). The reduction of the cell viability did not exceed 10% for 17 h, which supported the idea that mitoFluo could serve as an antibacterial agent suitable for treating gram-positive bacterial infections in humans. The IC50 of mitoFluo was estimated as 4 μM for Rko cells, which was similar to the earlier reported data [22] on the IC50 for transformed fibroblasts. Therefore, the therapeutic index (TI) measured as IC50/MIC was 8:1 for Rko. 

In summary, the present study revealed the high antibacterial potency of fluorescein alkyl ester derivatives (Figure 3). Based on the measurements of their uncoupling action on RLM (Figure 2), along with the data on their depolarizing activity in *B. subtilis* cells (Figure 4) and their inertness towards bacterial membrane integrity (Figure 5), the strong suppression of the bacterial growth by the fluorescein derivatives (Figure 3) could be predominantly attributed to the protonophoric uncoupling caused by these compounds. Small deviations from the correspondence between the depolarizing and antibacterial efficacies among these three compounds might be attributed to the presence of additional cellular targets for alkyl-triphenylphosphonium groups.

## Figures and Tables

**Table 1:** The susceptibility of various bacterial strains to the fluorescein derivatives: Minimal Inhibitory Concentration (MIC) measurements.

	mitoFluo MIC (µM)	tol-mitoFluo MIC (µM)	C_10_-FL MIC (µM)
*B. subtilis* Br151	0.5	0.25	2
*B. subtilis* PY79	0.5	0.25	2
*B. pumilus* NCTC 8241	0.5	0.25	2
*S. aureus*	0.6	0.3	2.4
*E. coli* W3110	>15	>15	>15

## Supplementary Materials

The following are available online at https://www.mdpi.com/2218-273X/10/2/309/s1, Scheme S1: Synthesis of tol-mitoFluo. Scheme S2: Synthesis of C10-FL. Figure S1: MALDI spectra of tol-mitoFluo. Figure S2: ESI-MS spectra of C10-FL.

## Abbreviations

C10-FL, fluorescein decyl ester; mitoFluo, fluorescein decyl(triphenyl)phosphonium ester; tol-mitoFluo, fluorescein decyl(tri-p-tolyl)phosphonium ester; TPP, triphenylphosphonium; Dis-C3(5), 3,3′-dipropylthiadicarbocyanine iodide; FCCP, carbonyl cyanide p-(trifluoromethoxy)phenyl hydrazone; CCCP, carbonyl cyanide m-chlorophenyl hydrazone; DNP, 2,4-dinitrophenol; MIC, minimal inhibitory concentration; RLM, rat liver mitochondria; FCS, fluorescence correlation spectroscopy.
