# Supplementary material for: Fluorescein Derivatives as Antibacterial Agents Acting via Membrane Depolarization
Source: Biomolecules. 2020 Feb 15;10(2):309. doi: 10.3390/biom10020309 (PMC7072581; doi:10.3390/biom10020309)
Supplement: Supplementary file 1 [file biomolecules-10-00309-s001.pdf]

Scheme S1: Synthesis of tol-mitoFluo.

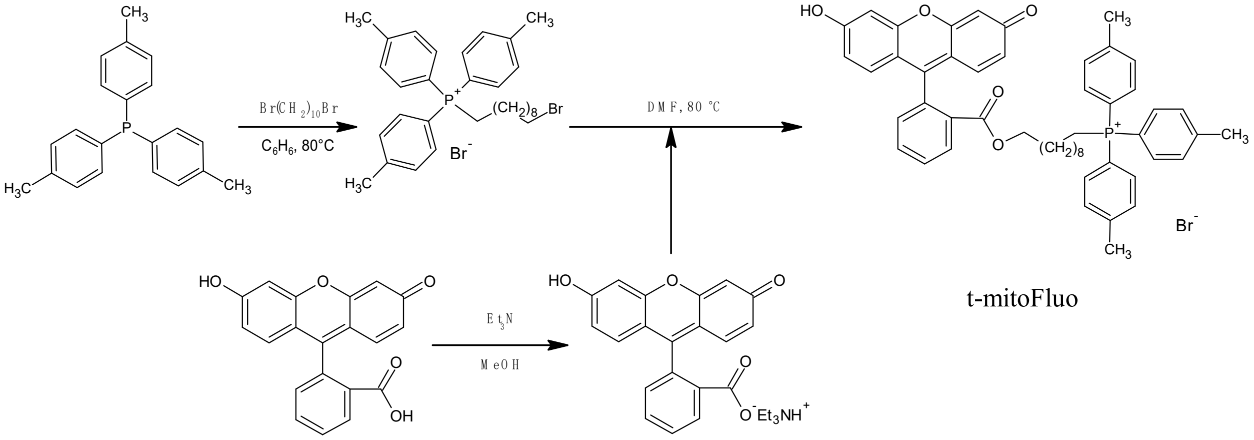

t-mitoFluo

Scheme S2: Synthesis of C10-FL.

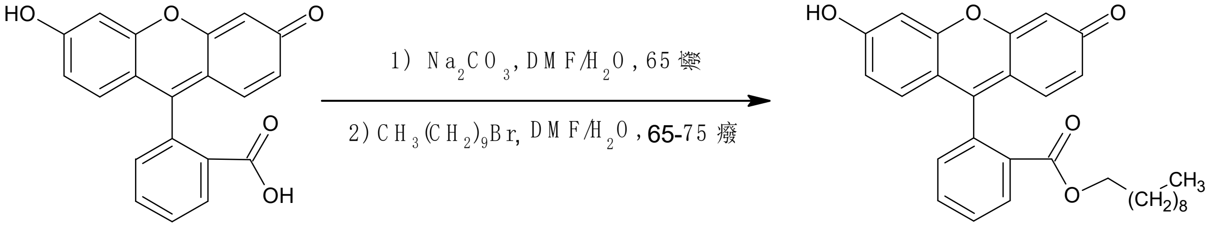

C10-FL

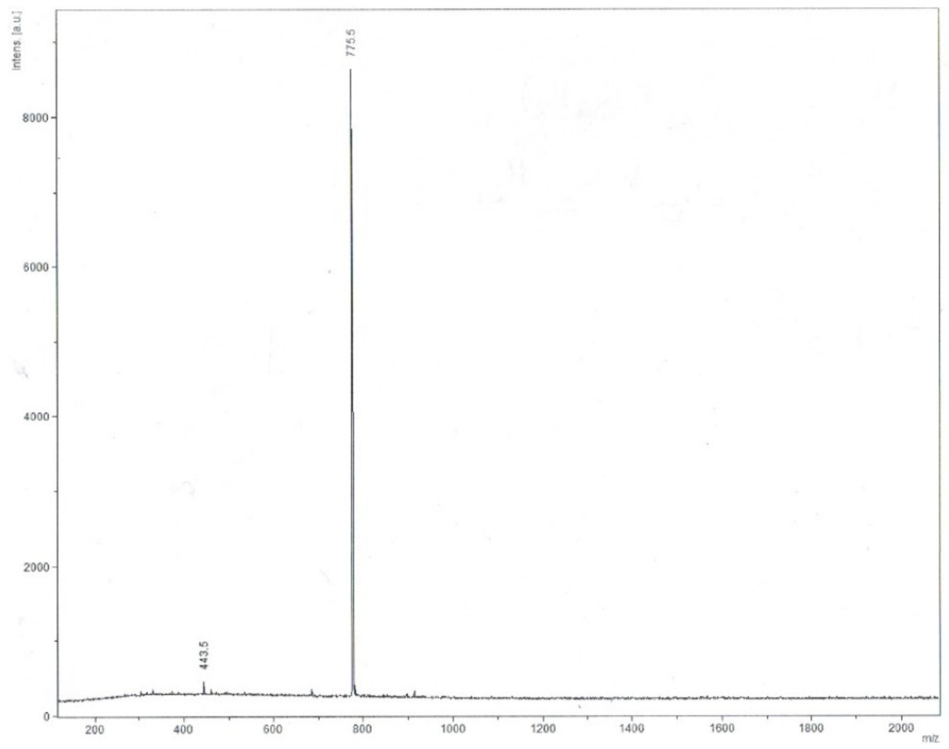

Figure S1. MALDI spectra of tol-mitoFluo.

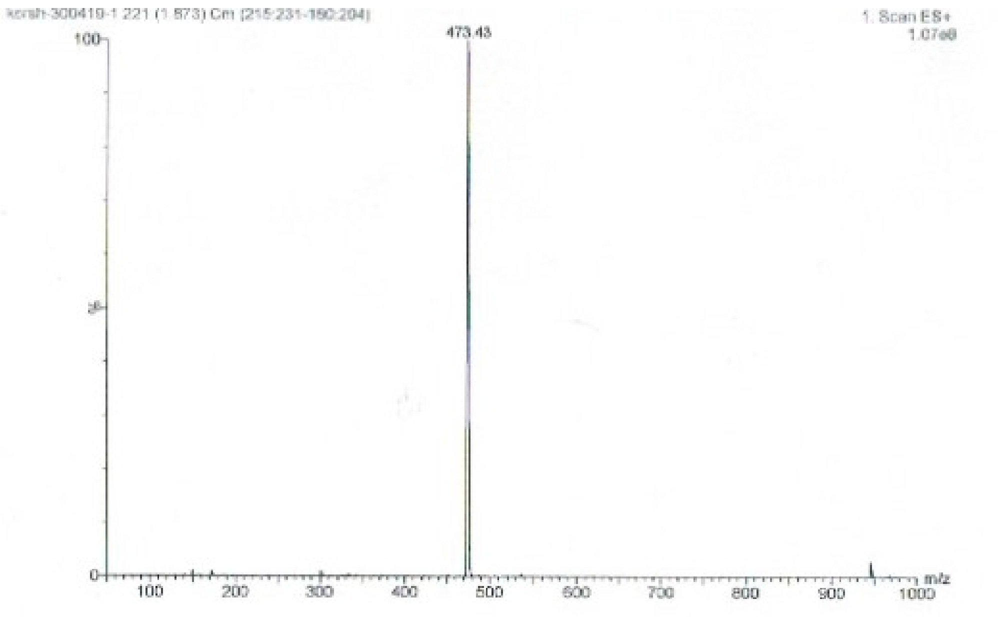

Figure S2. ESI-MS spectra of C10-FL.
